# Serotonergic Control of Metabolic Homeostasis

**DOI:** 10.3389/fncel.2017.00277

**Published:** 2017-09-20

**Authors:** Steven C. Wyler, Caleb C. Lord, Syann Lee, Joel K. Elmquist, Chen Liu

**Affiliations:** ^1^Division of Hypothalamic Research, Department of Internal Medicine, UT Southwestern Medical Center Dallas, TX, United States; ^2^Department of Pharmacology, UT Southwestern Medical Center Dallas, TX, United States; ^3^Department of Neuroscience, UT Southwestern Medical Center Dallas, TX, United States

**Keywords:** serotonin, obesity, diabetes, feeding, gluconeogenesis, adipose tissue, pancreas

## Abstract

New treatments are urgently needed to address the current epidemic of obesity and diabetes. Recent studies have highlighted multiple pathways whereby serotonin (5-HT) modulates energy homeostasis, leading to a renewed interest in the identification of 5-HT-based therapies for metabolic disease. This review aims to synthesize pharmacological and genetic studies that have found diverse functions of both central and peripheral 5-HT in the control of food intake, thermogenesis, and glucose and lipid metabolism. We also discuss the potential benefits of targeting the 5-HT system to combat metabolic disease.

## Introduction

A rich history of research connects serotonin (5-HT) signaling with food intake, energy expenditure, hormone balance and nutrient metabolism. Due to the growing public health crisis of obesity and diabetes, the role of 5-HT in metabolic homeostasis has recently led to a renewed interest in 5-HT pathways as novel therapeutic targets in the treatment of metabolic disease. 5-HT is synthesized from dietary l-tryptophan in a two-step enzymatic reaction involving tryptophan hydroxylases (TPHs) and l-amino acid decarboxylase (AADC; Walther and Bader, 2003). Centrally, 5-HT acts as a neurotransmitter produced in hindbrain raphe neurons that innervate virtually all brain regions (Jacobs and Azmitia, 1992; Muzerelle et al., 2016). Peripherally, 5-HT serves as a secreted hormone produced in intestinal enterochromaffin cells, enteric neurons, pancreatic cells, and adipose tissue (Berger et al., 2009). Since 5-HT does not cross the blood-brain barrier, peripheral and central 5-HT represent two distinct pools (Savelieva et al., 2008; Berger et al., 2009). The complexity of 5-HT signaling stems from a number of factors, including the large number of receptor genes (17 in humans and 14 in mice), alternative splicing of receptor transcripts, RNA editing of mRNA, varied combinations of receptor subunits, and heterodimerization with non-5-HT receptors (Hoyer et al., 2002; Lukasiewicz et al., 2010; Schellekens et al., 2015). All seven 5-HT receptor families (5-HT_1–7_) are G-protein coupled receptors, except for the 5-HT_3_ receptor, which is a pentameric, ligand-gated cation channel (Barrera et al., 2008). Several 5-HT receptors have been implicated in the regulation of metabolic homeostasis, including 5-HT_1B_, 5-HT_1F_, the 5-HT_2_ receptors (5-HT_2A-C_), 5-HT_3_ and 5-HT_6_ (Table 1) (Namkung et al., 2015; Voigt and Fink, 2015). In this review, we outline the roles of central and peripheral 5-HT signaling in metabolic homeostasis, and discuss the emerging therapeutic potential for targeting specific 5-HT receptors in the treatment of metabolic disease.

**Table 1:** Serotonergic receptors pertinent to this review.

Receptor	Signaling pathway	Tissue expression	Function
5-HT_1B_	G_i/o_	AgRP/NPY neurons	Suppress food intake
5-HT_1D_	G_i/o_	β-cell (pancreas)	Inhibits β-cell proliferation
5-HT_1F_	G_i/o_	α-cell (pancreas)	Inhibits glucagon secretion
5-HT_2A_	G_q/11_	White adipose	Inhibits lipolysis
			Promotes lipogenesis
5-HT_2B_	G_q/11_	White adipose	Promotes lipolysis
		β-cell (pancrease)	Promotes β-cell proliferation
			Promotes insulin secretion
		Liver	Promotes gluconeogenesis
			Inhibits hepatic glucose uptake
5-HT_2C_	G_q/11_	POMC/CART neurons	Suppress food intake
5-HT_3_	Cation channel	Brown adipose	Suppress BAT thermogenesis
		β-cell (pancrease)	Promotes insulin secretion
5-HT_6_	G_s_	PVN	Promotes food intake
5-HT_7_		IML	Modulates sympathetic outflow?

*Abbreviations: PVN, paraventricular nucleus; IML, Intermediolateral nucleus*.

## Central 5-HT Function in Metabolism

### Food Intake and Glucose Homeostasis

Over the past several decades, multiple studies have established a role for central 5-HT in reducing food intake and promoting satiety. For example, inhibition of central 5-HT synthesis with the TPH inhibitor, *para*-chlorophenylalanine (pCPA) or chemical lesion of 5-HT neurons with 5,7-dihydroxytryptamine (5,7-DHT) is orexigenic (Breisch et al., 1976; Saller and Stricker, 1976). Conversely, increasing synaptic 5-HT bioavailability, either by facilitating vesicular release with fenfluramine or by inhibiting 5-HT reuptake with selective serotonin reuptake inhibitors (SSRIs), produces an anorexigenic effect (Simansky and Vaidya, 1990; Heisler et al., 1997; Heal et al., 1998; Silverstein-Metzler et al., 2016). In addition to reducing food intake, increased central 5-HT signaling also improves glucose homeostasis, as treatment with fenfluramine or meta-chlorophenylpiperazine (mCPP, a 5-HT_1B/2C_ receptor agonist) improves glucose tolerance and insulin sensitivity (Storlien et al., 1989; Zhou et al., 2007).

A growing body of literature has explored the central 5-HT receptors that mediate 5-HT’s effect on food intake and glucose homeostasis. Of these receptors, the 5-HT_2C_ (G_q/11_ coupled) receptor has been the most studied. *Htr2c*^−/−^ mice develop late onset obesity with hyperphagia, and demonstrate a blunted anorectic response to fenfluramine and mCPP (Tecott et al., 1995; Nonogaki et al., 1998; Vickers et al., 1999; Xu et al., 2008). Moreover, these mice manifest hepatic insulin resistance, independent of body weight gain (Xu et al., 2008). Furthermore, loss of the 5-HT_2C_ receptor synergistically impairs glucose homeostasis in the diabetic *ob*/*ob* mouse model without exacerbating obesity (Wade et al., 2008). Of note, the excessive weight gain and increased risk of type 2 diabetes associated with atypical antipsychotic drugs (AATPs) may be due to antagonism of 5-HT_2C_ (Godlewska et al., 2009; Laika et al., 2010). Finally, the hyperphagia observed in Prader-Willi syndrome may be partially due to alterations in Htr2c mRNA splicing and editing that reduce the sensitivity of the 5-HT_2C_ receptor (Kishore and Stamm, 2006; Kawahara et al., 2008; Morabito et al., 2010; Garfield et al., 2016; Zhang et al., 2016).

**Figure 1:**
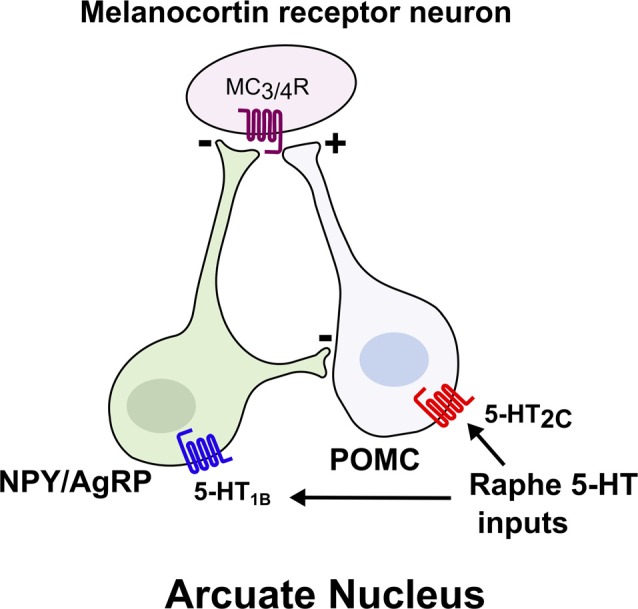
Brain 5-HT acts on central melanocortin neurons to suppress food intake. Melanocortin receptor (MC_3/4_R) neurons integrate signals from two reciprocal populations of neurons within the arcuate nucleus to promote satiety. Activation of 5-HT_2c_ increases the activity of anorexigenic proopiomelanocortin (POMC) neurons, whereas activation of 5-HT_1B_ inhibits the orexigenic NPY/AgRP neurons.

Several recent studies have also shed light on the neural circuits that mediate central 5-HT’s effects on energy balance, most notably the central melanocortin system, which includes two reciprocal populations of melanocortin neurons within the arcuate nucleus of the hypothalamus (ARC), anorexigenic neurons expressing proopiomelanocortin (POMC) and orexigenic neurons expressing neuropeptide Y/Agouti related peptide (NPY/AgRP; Figure 1; Sohn et al., 2013). Melanocortin receptors (MC_3_R and MC_4_R) in downstream neurons such as the paraventricular nucleus (PVN) are activated by alpha-melanocyte stimulating hormone (α-MSH), a proteolytic product of POMC, and inhibited by AgRP to reciprocally regulate food intake and glucose homeostasis (Berglund et al., 2014; Garfield et al., 2015; Krashes et al., 2016). Approximately 25% of POMC neurons in the adult mouse brain functionally express 5-HT_2C_ receptors (Xu et al., 2010b; Sohn et al., 2011). 5-HT_2C_ receptor activation in POMC neurons both induces *Pomc* mRNA expression and increases POMC neuronal activity through activation of TRPC5 cation channels (Zhou et al., 2007; Lam et al., 2008; Xu et al., 2010b; Gao et al., 2017). Remarkably, re-expression of 5-HT_2C_ only in POMC neurons in an otherwise *Htr2c*^−/−^ mouse is sufficient to reverse the hyperphagia and liver insulin resistance characteristic of *Htr2c* deficiency (Xu et al., 2010a,b). Conversely, mice with a POMC neuron-specific deletion of *Htr2c* are hyperphagic, show a blunted anorectic response to fenfluramine or mCPP, and have impaired glucose homeostasis (Berglund et al., 2013). Together, these studies underscore a critical role for 5-HT_2C_ in POMC neurons to regulate food intake and hepatic glucose metabolism.

In addition to 5-HT_2C_, the 5-HT_1B_ receptor has been implicated in body weight regulation. Agonists for 5-HT_1B_ suppress food intake (Lee and Simansky, 1997), whereas genetic ablation of *Htr1b* leads to increased food intake and body weight (Halford and Blundell, 1996; Lee et al., 1998; Lucas et al., 1998). Moreover, the anorectic effect of fenfluramine is blunted in *Htr1b* null mice (Lucas et al., 1998). Collectively, these findings suggest that *Htr1b* contributes to 5-HT’s effect on food intake and body weight. Activation of 5-HT_1B_ seemingly inhibits NPY/AgRP neurons to promote satiety, as administration of 5-HT or a 5-HT_1B_ agonist hyperpolarizes NPY/AgRP neurons and subsequently reduces inhibitory postsynaptic currents (IPSCs) on POMC neurons (Figure 1; Heisler et al., 2006). Thus, 5-HT appears to promote satiety by simultaneously activating anorexigenic POMC neurons and inhibiting orexigenic AgRP/NPY neurons, due to the expression of 5-HT_2C_ and 5-HT_1B_, respectively (Heisler et al., 2006). As a result, the reciprocal functions of these two receptors synergize to promote the activation of downstream melanocortin receptor-expressing neurons. Indeed, it has been shown that intact MC_4_R signaling is required for the anorectic effects of 5-HT compounds (Heisler et al., 2002, 2003, 2006; Lam et al., 2008; Xu et al., 2010b).

Less is known about the role of 5-HT_6_ (G_s_ coupled) receptors in food intake and satiety. Most of the current evidence comes from pharmacological studies using 5-HT_6_ receptor agonists and antagonists, as well as *Htr6* siRNA experiments (Woolley et al., 2001; Dudek et al., 2015; Higgs et al., 2016). Unlike 5-HT_2C_ and 5-HT_1B_ receptors, where activation leads to hypophagia, antagonism of 5-HT_6_ reduces food intake and promotes satiety (Heal et al., 2008; Dudek et al., 2015; Higgs et al., 2016; Oh et al., 2016). Consistent with this, *Htr6*^−/−^ mice are partially protected from diet-induced obesity due to reduced food intake (Frassetto et al., 2008). Although the metabolically relevant neuronal circuits modulated by 5-HT_6_ receptors remain unclear, a recent study mapping 5-HT_6_ antagonist-induced c-Fos activity suggests that the PVN is an important site of action (Garfield et al., 2014). A potential role for 5-HT_6_ in energy expenditure and glucose homeostasis warrants further study.

### Thermoregulation

**Figure 2:**
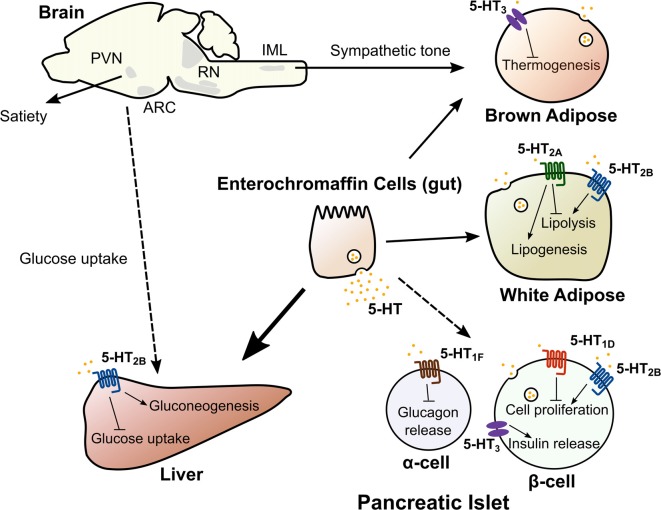
Central and peripheral 5-HT acts on multiple organs to regulate metabolic homeostasis. 5-HT in the central nervous system acts on multiple nodes of the neuroaxis (hypothalamus, brain stem and the spinal cord) to regulate satiety, hepatic glucose uptake and adaptation to cold exposure. Dashed line from brain to liver indicates the mechanism regulating hepatic glucose uptake is unknown. Peripheral 5-HT produced by intestinal enterochromaffin cells, pancreatic islets and adipose tissue exerts local and/or systemic control of lipid and glucose homeostasis though distinct 5-HT receptors. Abbreviations: ARC, Arcuate nucleus; PVN, Paraventricular nucleus; RN, Raphe nuclei; IML, Intermediolateral nucleus.

Thermogenesis through mitochondrial uncoupling occurs primarily in brown adipose tissue (BAT) and in beige adipocytes, a type of thermogenic adipocyte that appears in subcutaneous white adipose tissue (WAT) during prolonged cold exposure (Cohen and Spiegelman, 2015). Adaptive thermogenesis has recently gained widespread attention as a potential therapy to combat obesity by increasing energy expenditure. Central 5-HT appears to promote thermogenesis, since pharmacological or genetic depletion of central 5-HT has been found to impair thermogenic adaptation to cold (Fuller et al., 1987; Alenina et al., 2009; Hodges et al., 2011; Cerpa et al., 2014; McGlashon et al., 2015). For instance, mice with central depletion of 5-HT have reduced adaptation to cold exposure, diminished thermogenic function of BAT, and decreased recruitment of beige adipocytes (Alenina et al., 2009; Hodges et al., 2011; Cerpa et al., 2014; McGlashon et al., 2015). Central 5-HT appears to increase BAT and beige adipocyte thermogenic function by modulating sympathetic outflow to these tissues. Transynaptic retrograde tracing from BAT synaptic terminals show that glutamatergic and 5-HT neurons of the rostral raphe pallidus synapse onto sympathetic fibers in the intermediolateral nucleus (IML) of the spinal cord (Figure 2; Bowker et al., 1981; Bamshad et al., 1999). Systemic or IML injections of 5-HT or fenfluramine increase sympathetic firing of these fibers, while IML injections of 5-HT_7_ receptor antagonists decrease sympathetic tone (Arase et al., 1988; Madden and Morrison, 2006, 2010; Morrison, 2016). Collectively, these data suggest that inputs from central 5-HT pathways play a significant role in the adaptation to cold exposure through the sympathetic activation of thermogenic adipose tissue.

## Peripheral 5-HT Function in Metabolism

The vast majority (over 95%) of 5-HT in the body is produced outside the nervous system (Berger et al., 2009). Tryptophan hydroxylase 1 (TPH1) is the main TPH isoform responsible for 5-HT synthesis in the periphery. Knockout of *Tph1* in mice has a minor effect on brainstem 5-HT levels, but leads to an almost complete loss of intestinal and blood 5-HT (Côté et al., 2003; Izikki et al., 2007; Savelieva et al., 2008). Peripheral 5-HT has been mainly studied for its role in gut motility, immunology, and cardiovascular function (Côté et al., 2004; Gershon and Tack, 2007; Duerschmied and Bode, 2009; Shajib and Khan, 2015). However, accumulating evidence suggests that peripheral 5-HT also acts an endocrine factor to regulate metabolic function in multiple tissues (El-Merahbi et al., 2015).

### 5-HT in Pancreas

Pancreatic islets, or islets of Langerhans, contain hormone-secreting endocrine cells. The two major endocrine cells in pancreatic islets are the beta cells, which secrete insulin, and the alpha cells, which secrete glucagon. In the fed state, insulin inhibits endogenous glucose production and stimulates glucose uptake and conversion into glycogen and lipid. Conversely, glucagon acts to increase blood glucose levels during fasting by stimulating glycogenolysis and gluconeogenesis. Insulin also acts on the alpha cells to inhibit glucagon secretion (Cooperberg and Cryer, 2010).

5-HT is synthesized in pancreatic islets and co-secreted along with insulin, potentially acting as a local autocrine/paracrine signal (Ekholm et al., 1971; Lundquist et al., 1971; Gylfe, 1978; Richmond et al., 1996; Ohta et al., 2011; Almaca et al., 2016; Bennet et al., 2016). 5-HT appears to be required for normal insulin secretion, since the loss of 5-HT synthesis within beta cells impairs insulin secretion (Paulmann et al., 2009; Kim et al., 2015). Consistent with this, posttranslational modification with 5-HT (known as serotonylation) activates the small GTPases Rab3a and Rab27a in the insulin secretion pathway, leading to increased insulin exocytosis (Paulmann et al., 2009). In addition, activation of 5-HT_2B_ receptor in isolated pancreatic islets has been found to augment glucose-stimulated insulin secretion (Bennet et al., 2016). Notably, deletion of *Tph1* in intestinal enterochromaffin cells in mice does not alter insulin secretion, which further supports the notion that locally synthesized 5-HT in the pancreas serves as an autocrine signal to support normal insulin secretion (Sumara et al., 2012). In addition to regulating insulin secretion, there is evidence that 5-HT also regulates glucagon secretion (Marco et al., 1977; Adeghate et al., 1999). For example, human islet beta cells reportedly release physiological levels of 5-HT in response to glucose (Almaca et al., 2016). The release of 5-HT significantly decreased glucagon secretion, and this effect was mediated by the 5-HT_1F_ (G_i/o_ coupled) receptor on alpha cells. Interestingly, a 5-HT_1F_ receptor agonist, LY344864, was able to inhibit glucagon release from human islets. Systemic administration of this drug reduced plasma glucagon levels in hypoglycemic mice, suggesting that this pathway is active physiologically.

Pancreatic 5-HT may also play an important role under conditions of metabolic stress. For example, it has been suggested that islet 5-HT functions during pregnancy to increase beta cell mass and glucose-stimulated insulin secretion (Kim et al., 2010; Ohara-Imaizumi et al., 2013). Very early in pregnancy, the islet cell expression of *Tph1* and *Tph2* increases, resulting in a 400-fold increase in 5-HT levels (Kim et al., 2010). This 5-HT may augment glucose-stimulated insulin secretion in beta cells through activation of the ligand-gated cation channel 5-HT_3_ receptor, increasing beta cell depolarization in response to glucose. Moreover, 5-HT signaling through 5-HT_2B_ (G_q_ coupled) has been implicated in pregnancy-induced maternal beta cell proliferation, which is later reversed by upregulation of the 5-HT_1D_ receptor (G_i/o_ coupled) in the perinatal period (Kim et al., 2010). In addition to its adaptive role in pregnancy, pancreatic 5-HT also appears to influence the metabolic adaptation to conditions of high fat diet-induced obesity and insulin resistance. In mice fed a high fat diet, the beta cell-specific loss of either *Tph1* or the *Htr3a* receptor subunit results in impaired glucose tolerance (Kim et al., 2015). In addition, there is a positive correlation between body mass index (BMI) and the number of 5-HT immunoreactive cells in the human pancreas, further supporting an role for pancreatic 5-HT signaling during metabolic stress (Almaca et al., 2016).

### 5-HT in Liver

In the fed state, the liver converts excess glucose into glycogen and activates *de novo* lipid synthesis. Conversely, in the fasted state, the liver liberates glycogen stores, produces glucose through *de novo* synthesis (gluconeogenesis), and generates ketone bodies from fatty acids. Studies examining the effect of 5-HT on hepatic gluconeogenesis, glycogen storage, glucose uptake, and glycolysis have produced conflicting results, likely due to differences in routes of 5-HT administration and the discrepancy between *in vivo* and *in vitro* models (Zabala et al., 1992; Moore et al., 2004a,b; An et al., 2009; Watanabe et al., 2010; Tudhope et al., 2012; El-Merahbi et al., 2015). However, Sumara et al. recently used genetic models to elegantly show that circulating 5-HT synthesized in intestinal enterochromaffin cells can signal through the 5-HT_2B_ receptor in hepatocytes to stimulate liver gluconeogenesis and inhibit glucose uptake by the liver during the fasted state (Sumara et al., 2012). In addition, 5-HT has been shown to regulate hepatic bile acid turnover and lipid metabolism (Watanabe et al., 2010). 5-HT treatment in mice accelerated the turnover of bile acids (excretion by the gallbladder followed by reabsorption by the intestine) and increased the concentration of circulating bile acids. 5-HT treatment also decreased liver triglyceride levels and increased liver cholesterol levels. However, it remains unclear whether this reflects a physiological role of 5-HT in hepatic lipid metabolism. In addition, systemic deficiency or chemical inhibition of TPH1 in high fat diet-fed mice reduced hepatic steatosis, secondary to decreased body weight and increased adipose thermogenesis (Crane et al., 2015). Thus, a direct link between physiological levels of 5-HT and hepatic lipid metabolism is not as clear as the role of 5-HT in hepatic glucose metabolism. Nevertheless, these studies overall suggest that gut-derived 5-HT plays an important role in the liver’s control of metabolic homeostasis.

### 5-HT in Adipose Tissue

Adipose tissue is a complex organ with multiple depots. WAT stores excess energy as triglycerides and releases non-esterified fatty acids (NEFA) and glycerol through lipolysis during fasting. WAT also acts as a major endocrine organ by secreting key hormones, including leptin and adiponectin, which regulate systemic metabolic homeostasis. There is evidence that both gut-derived circulating 5-HT and adipocyte-derived 5-HT play important roles in adipose tissue function. It has been known for decades that 5-HT administration increases circulating NEFA and glycerol levels (Carlson et al., 1967; Sumara et al., 2012). Fasting increases circulating 5-HT levels, and intestine-specific deletion of *Tph1* in mice blunts fasting-induced plasma glycerol and NEFA levels (Sumara et al., 2012). 5-HT has also been implicated in adipose tissue lipogenesis. Recently, the recruitment of beige adipocytes in subcutaneous WAT, also known as browning of WAT, has generated widespread interest as a potential target for treating obesity (Cohen and Spiegelman, 2015). Interestingly, pharmacological inhibition of 5-HT synthesis protects mice from high fat diet-induced obesity through decreased adipose tissue lipogenesis, increased browning in subcutaneous WAT, and increased BAT thermogenesis (Crane et al., 2015; Oh et al., 2015). Of note, it appears that central 5-HT increases adipose tissue thermogenesis, whereas peripheral 5-HT inhibits it, highlighting the distinct roles of central and peripheral 5-HT. Interestingly, 5-HT appears to be locally synthesized as an autocrine factor in adipocytes, since adipocyte-specific deletion of *Tph1* results in a similar phenotype as the systemic loss of *Tph1* (Oh et al., 2015). Collectively, this evidence suggests that peripheral 5-HT stimulates adipose tissue lipolysis during fasting, promotes lipogenesis in adipose tissue in response to high fat diet, and inhibits adaptive thermogenesis. Specific 5-HT receptors have been implicated in each of these functions. 5-HT modulation of lipolysis appears to be mediated by the 5-HT_2B_ receptor, since adipose deletion of *Htr2b* results in blunted fasting-induced lipolysis and nearly complete ablation of serotonin-induced lipolysis (Sumara et al., 2012). Treatment with a 5-HT_2A_ receptor antagonist blocks lipid accumulation in 3T3-L1 adipocytes (Oh et al., 2015). Finally, *Htr3a* knockout mice exhibit increased thermogenesis and reduced weight gain (Oh et al., 2015).

It is interesting to note the reported major source of 5-HT in each of the metabolic tissues discussed here: islet-derived 5-HT in the pancreas, gut-derived 5-HT in the liver, and a combination of gut-derived 5-HT and adipocyte-derived 5-HT in adipose tissue. These differences, combined with the large number of 5-HT receptors, highlight the complexity of the peripheral serotonin system and the need for continued work to better understand the role of serotonin in metabolism.

## Conclusion/Clinical Significance

These wide-ranging effects of 5-HT in metabolism have driven a renewed interest in identifying 5-HT-related therapeutics for metabolic disease. 5-HT-based strategies include either altering bulk 5-HT bioavailability or targeting individual or groups of 5-HT receptors. Presently, the only 5-HT-based drug approved for treatment of obesity is lorcaserin, which selectively targets the 5-HT_2C_ receptor.

SSRIs, which increase postsynaptic 5-HT bioavailability, are known to reduce weight in animal studies (Heisler et al., 1997; Silverstein-Metzler et al., 2016), In particular, an intriguing 18 month longitudinal study in macaques suggested that SSRI treatment decreased adiposity and improved insulin sensitivity (Silverstein-Metzler et al., 2016). However, the metabolic effects of SSRIs are less clear in human, with some studies showing weight gain and others showing weight loss (Simansky and Vaidya, 1990; Heisler et al., 1997; Heal et al., 1998; Silverstein-Metzler et al., 2016). These studies must be interpreted with caution due to the comorbidity of anxiety/depressive disorders with metabolic diseases. We are unaware of any metabolic studies of SSRI treatment in healthy individuals.

Due to the potential for off-target effects of altering bulk 5-HT levels, targeting specific 5-HT receptors is likely to be a better strategy. The 5-HT_2C_ receptor remains a tantalizing target for treatment of obesity. The weight loss effects of fenfluramine appear to result largely through the 5-HT_2C_ receptor (Xu et al., 2010a,b). Recently, the 5-HT_2C_ receptor agonist lorcaserin (Belviq^®^) was approved by the FDA for the treatment of obesity (Smith et al., 2010). In addition to combating obesity in the general population, another potential use of 5-HT_2C_ agonists could be to treat metabolic syndrome in patients taking AATPs. AATPs such as olanzapine and clozapine frequently induce increased food intake, excessive weight gain and higher risk of diabetes (MacNeil and Müller, 2016). Genetic variants in the 5-HT_2C_ receptor have been associated with susceptibility to the adverse effects of AATPs (Godlewska et al., 2009; Laika et al., 2010), and these adverse effects may be due to off-target antagonism of 5-HT_2C_ by AATPs (Wallace et al., 2011; MacNeil and Müller, 2016). Given that the psychotropic effects of AATPs are thought to be mediated primarily by the dopaminergic system, it is plausible that a 5-HT_2C_ receptor specific agonist such as lorcaserin could be used to alleviate the untoward metabolic side effects of AATPs, without interfering with the antipsychotic benefits. In addition, since 5-HT_2C_ and 5-HT_1B_ synergistically activate melanocortin neurons to promote satiety, combined treatment with 5-HT_2C_ and 5-HT_1B_ receptor agonists may have a greater anorexigenic effect than 5-HT_2C_ agonists alone (Heisler et al., 2006; Doslikova et al., 2013). The 5-HT_6_ receptor is another potential target, given the possibility that antagonists such as idalopirdine, which recently passed phase I and phase II studies for the treatment of Alzheimer’s disease, could also be effective for the treatment of obesity (Dudek et al., 2015; Ferrero et al., 2017). Finally, in light of the proposed inhibitory role for 5-HT_1F_ in glucagon secretion, the 5-HT_1F_ receptor agonist, Lasmiditan, which is currently in Phase III clinical trials for migraines, could be a potential therapeutic target to suppress glucagon release in diabetics (Reuter et al., 2015; Almaca et al., 2016; Barbanti et al., 2017).

In conclusion, although 5-HT plays a clear role in metabolic regulation, further work is needed to understand the complex biology arising from the large number of 5-HT receptors and their relative contributions to central and peripheral energy homeostasis. Through the continued increase in human genetic data, the identification of functional rare genetic variants in humans combined with genetic animal models should greatly advance our understanding of 5-HT in metabolic signaling, enabling novel targeted therapies for obesity and diabetes.

## Author Contributions

SCW and CCL: wrote the manuscript. SL, JKE and CL edited the manuscript.

## Conflict of Interest Statement

The authors declare that the research was conducted in the absence of any commercial or financial relationships that could be construed as a potential conflict of interest.
